# Therapist Effects and the Impact of Early Therapeutic Alliance on Symptomatic Outcome in Chronic Fatigue Syndrome

**DOI:** 10.1371/journal.pone.0144623

**Published:** 2015-12-14

**Authors:** Lucy P. Goldsmith, Graham Dunn, Richard P. Bentall, Shôn W. Lewis, Alison J. Wearden

**Affiliations:** 1 Centre for Biostatistics, Institute of Population Health, University of Manchester, Manchester, United Kingdom; 2 Manchester Academic Health Science Centre, Manchester, United Kingdom; 3 School of Health and Human Sciences, University of Huddersfield, Huddersfield, United Kingdom; 4 Institute of Psychology, Health and Society, University of Liverpool, Liverpool, United Kingdom; 5 Institute of Brain, Behaviour and Mental Health, University of Manchester, Manchester, United Kingdom; 6 School of Psychological Sciences and Manchester Centre for Health Psychology, University of Manchester, Manchester, United Kingdom; University of Marburg, GERMANY

## Abstract

Few studies have examined therapist effects and therapeutic alliance (TA) in treatments for chronic fatigue syndrome (CFS). Therapist effects are the differences in outcomes achieved by different therapists. TA is the quality of the bond and level of agreement regarding the goals and tasks of therapy. Prior research suffers the methodological problem that the allocation of therapist was not randomized, meaning therapist effects may be confounded with selection effects. We used data from a randomized controlled treatment trial of 296 people with CFS. The trial compared pragmatic rehabilitation (PR), a nurse led, home based self-help treatment, a counselling-based treatment called supportive listening (SL), with general practitioner treatment as usual. Therapist allocation was randomized. Primary outcome measures, fatigue and physical functioning were assessed blind to treatment allocation. TA was measured in the PR and SL arms. Regression models allowing for interactions were used to examine relationships between (i) therapist and therapeutic alliance, and (ii) therapist and average treatment effect (the difference in mean outcomes between different treatment conditions). We found no therapist effects. We found no relationship between TA and the average treatment effect of a therapist. One therapist formed stronger alliances when delivering PR compared to when delivering SL (effect size 0.76, SE 0.33, 95% CI 0.11 to 1.41). In these therapies for CFS, TA does not influence symptomatic outcome. The lack of significant therapist effects on outcome may result from the trial’s rigorous quality control, or random therapist allocation, eliminating selection effects. Further research is needed.

***Trial Registration*:** ISRCTN74156610

## Introduction

Chronic Fatigue Syndrome (CFS), also known as myalgic encephalomyelitis or encephalopathy (ME), is characterised by severe, disabling fatigue which affects both physical and mental functioning [1]. The most effective treatments for CFS are cognitive behaviour therapy (CBT) and graded exercise therapy (GET) delivered by expert therapists [2–4]. Pragmatic rehabilitation, an approach which combines elements of CBT and GET, delivered by trained general nurses was effective in improving fatigue over an 18-week treatment period but effects attenuated over one year follow up [5].

Identifying the components of therapy which cause symptomatic change aids theoretical understanding of the processes underlying therapeutic change, can improve current practice, and supports the development of more effective therapies [6]. Several studies have examined mechanism of change in treatment trials for CFS. A reduction in fear related avoidance of activity has been shown to mediate the effects of both CBT and GET on fatigue[7], as has a decrease in focusing on symptoms[8–10] and an increased sense of control over fatigue[9]. Wearden and Emsley [11] showed that reduction in fatigue after pragmatic rehabilitation was mediated by changes in cognitive and behavioural responses to fatigue, namely reductions in catastrophizing and self-reported activity limitation. However, a limitation of prior work on mediators of change is that potential confounding between purported mediators and outcome has not been addressed, meaning that the purported mediators may merely correlate with, rather than *cause* the improvement in outcome [12].

In any psychological intervention, it may be difficult to decide whether outcomes are determined by specific techniques or mechanisms such as those described above, or, alternatively, by more general psychological processes such as the therapeutic alliance [13]. It may also be unclear whether patient or therapist characteristics most determine outcome. Here, will consider two therapist factors which might be related to outcome, therapist effects and therapeutic alliance.

“Therapist effects” refer to the tendency of different therapists to obtain differential symptomatic change in the patient. Therapists often have allegiances to therapeutic approaches or superior skills in delivering one particular therapy[14–16], and allegiance effects can be sizeable[16–18]. Reviewing research across all psychological disorders and interventions, Crits-Christoph and Mintz [19] found that better controlled outcome studies have smaller therapist effects, suggesting that quality control procedures (manualized therapy, careful selection, training and supervision of therapists) are successful in minimizing therapist variation. They estimated that therapist effects account for 0% to 50% of change in symptomatic outcome.

To date, three studies have examined therapist effects in chronic fatigue syndrome, with contrasting results. Two studies used data collected in routine care (not controlled treatment trials). Wiborg *et al*. [20] found therapist effects accounted for 21% of variance in post-treatment fatigue. Cella *et al*. [21] found the therapist effect on outcome accounted for 0% for fatigue and under 2% for disability, while Godfrey *et al*. [22], analysing data from a treatment trial of CBT versus counselling for chronic fatigue, found no detectable therapist effect on outcome. Wiborg’s finding [20] could be explained by therapists’ attitudes towards a manualized approach and a centre effect, while Cella’s study [21]took place in a single specialist centre, using therapists of the same orientation and training, and a shared environment and supervision for the therapists. In none of these studies were patients randomly assigned to therapists.

Therapeutic alliance refers to the quality of the relationship between therapist and patient and is commonly accepted to have three components: task, goal and bond [23]. Goals refer to what the patient hopes to gain from therapy. Tasks are what the patient and therapist jointly agree need to be completed to reach the patient's goals. The bond forms from trust and confidence that the tasks will help the patient achieve the goals. Meta-analytic reviews of research with patients across a range of therapies and diagnoses consistently claim a good alliance predicts positive outcome across different therapy modalities and different conditions, with moderate effect sizes [24–26].

Two studies have examined therapeutic alliance in CFS. Heins *et al*. [27]investigated the effects of the therapeutic alliance on fatigue, using a definition of the therapeutic relationship given by Wampold and Budge [28] that included outcome expectations as well as Bordin’s [23] three components of task, goal and bond. Regressing symptomatic outcome on baseline variables and the therapeutic relationship, Heins *et al*. [27] reported that 25% of the variance in post-treatment fatigue was explained by the therapy relationship as rated by the patient. However, their reported results (their Table 2) show that that the bond and goal components of therapeutic alliance did not significantly contribute to the model. The task element of alliance contributed significantly, but the patients’ expectations explain the most variance. A methodological limitation of the study was that it used mean imputation to replace missing outcome data, potentially introducing bias [29]. Godfrey *et al*. [22] conducted a principal component analysis of fourteen items measuring therapeutic alliance in the context of a randomized controlled trial of CBT vs counselling delivered by six therapists. Three factors emerged which were labelled working strategy consensus, therapist contribution and patient contribution. Of these, only patient contribution significantly correlated with fatigue at 6 months. Subsequent analysis suggested that a non-alliance measure, ‘emotional processing’, was significantly associated with a good outcome.

The present paper examines therapist effects and the effect of therapeutic alliance in a secondary analysis of a randomized controlled trial of treatments for patients with chronic fatigue syndrome, which has been described and reported elsewhere[5, 30]. The two treatments are pragmatic rehabilitation and supportive listening, a non-directive counselling based treatment. Pragmatic rehabilitation was first reported in 2001 [31]. It is an educational self-help treatment for CFS/ME which starts by providing patients with a detailed explanatory model for their symptoms, supported by a patient manual containing references to the evidence base. While pragmatic rehabilitation has aspects in common with cognitive behavioural therapy and graded exercise therapy, unlike those therapies, it was designed to be delivered by non-specialists who have been taught the explanatory model. After the presentation of the model, the programme of rehabilitation is worked out collaboratively by the patient and the therapist, and is tailored to the patient’s specific goals, but it always includes a programme of graded increases in activity.

To enable the present study to be comparable to the larger body of research in psychological therapies across diagnoses, we use the common definition of therapeutic alliance as including the task, goal and bond. To enable comparison to the work of Heins *et al*. [27], the present study also examines the effect of the task element of alliance separately.

## Method

Patients in the FINE Trial were randomized to one of three treatment arms: pragmatic rehabilitation (PR) or supportive listening (SL), each delivered with treatment as usual from the general practitioner (TAU), or TAU alone. The two active therapies (PR and SL) were delivered by three general nurses specially trained for the study, with all three nurses delivering both therapies. The three nurses, all female, were all experienced registered nurse practitioners who had worked in primary care settings for many years, but who did not have a specialist interest in CFS prior to their employment on the trial. They were not qualified CBT therapists and had not delivered psychological therapy of this type previously. The nurses received training over a six month period in research on CFS, the delivery of the interventions, and working to a protocol. Training involved supervised practice, role play and discussions. At the end of training the nurses’ competence to deliver the interventions was assessed as satisfactory on the basis of their performance in the practice therapy sessions. The therapists received regular supervision during the delivery of the interventions. Fidelity to the treatment approaches was assessed from randomly selected audiotapes of therapy sessions and was judged to be good by independent raters.

Randomization was carried out by an independent telephone randomization service based at the Christie Hospital Clinical Trials Unit in Manchester, using randomised permuted blocks (with randomly-varying block sizes of 9, 12, 15 or 18), after stratification on two variables—whether or not the patient was non-ambulatory, defined as using a mobility aid on most days, and whether or not the patient fulfilled the London criteria for a diagnosis of ME [5, 30]. The purpose of stratification on ambulatory status and London ME criteria was in order to address the possibility that those with a higher degree of disability and those who fulfilled the London ME criteria might be different from the rest of the sample. A randomized assignment of therapist was employed, with patients assigned to therapists in a simple random fashion constrained only by the availability of the therapists.

One hundred and ninety six participants, aged 18 and over and diagnosed with CFS/ME using the Oxford criteria [1], were randomized, 95 to PR and 101 to SL. The remaining 100 were randomized to TAU alone, but the TAU group is not included in the current analysis, as these patients were simply continuing standard contact with their GPs and did not meet a therapist but, more importantly, because they are a common control group for all three therapists, their data do not contribute any information concerning treatment effect heterogeneity arising from therapist effects. Patients were excluded from the FINE Trial if they fulfilled diagnostic criteria for antisocial, borderline, or paranoid personality disorders, had active suicidal ideation, were unable to read or write English, were currently undertaking systematic psychological therapies for CFS/ME, or had received pragmatic rehabilitation in the past year. Further details of the FINE trial sample and procedures, and a figure showing participant progression through the study are available in the main trial protocol and write-up [5, 30]. The FINE study was granted ethical approval by Eastern MREC (03/05/62). The data were anonymized prior to analysis. As a secondary analysis of an anonymized and de-identified clinical trial dataset, no further ethical approval was required for this study.

### Measures

#### Patient Therapeutic Alliance Measure

A brief version of the California Psychotherapeutic Alliance Scale (CALPAS) [32] was completed by patients one week and 10 weeks after the start of therapy. Patients were asked to complete their forms confidentially at home and return them to the trial office by post following completion. The scale measured alliance using 5 items, each rated for agreement on an 11 point scale ranging from 0 (not at all) -100 (very much). Items asked about the therapists’ desire to understand the patient, therapist-patient agreement on goals, whether the treatment matched the patient’s ideas about helpful treatment approaches, joint working with the therapist and satisfaction with services received. The scale total score was the mean score on the five items. Here we present findings from the one week CALPAS measure only. We also analysed one of the items, measuring task alliance, separately, to enable comparison with other studies.

#### Outcome measures

Primary outcomes in the FINE Trial were self-reported physical functioning, measured using the 8-item physical functioning scale of The Short Form (36) Health Survey (SF-36) [33] and fatigue, measured using the Chalder fatigue scale [34]. In accordance with usual practice, SF-36 physical functioning scores were calculated from answers to ten questions about how much the participants health limited their activities, each question scored 0 (limited a lot), 1 (limited a little), or 2 (not limited at all), then summed and converted to a percentage. The 11 items of the Chalder fatigue scale ask about problems with physical and mental fatigue over the past three weeks, and have a response format “less than usual, no more than usual, more than usual and much more than usual”. Each item may be scored—either 0011 or 0123, and then the items summed to obtain a total fatigue scale score [35, 36]. When scored 0011, a score of 4 or more indicated clinically significant levels of fatigue [34]. Both methods of scoring were used in the present study. Both primary outcomes were assessed at the end of treatment and one year following the end of treatment (20 and 70 weeks).

#### Statistical approach

Change scores were calculated for both primary outcomes (fatigue and physical functioning) by subtracting 20 and 70 week scores from baseline scores from). First, we examined simple descriptive statistics, by therapist and therapy type (PR vs SL) for both outcomes and measures of the therapeutic alliance. Since (as a result of the trial design) the controls are common to all six therapist by therapy type combinations, differences in the efficacy of these six combinations can be compared without reference to the controls. For example, the effect of pragmatic rehabilitation when delivered by therapist 1 is the difference in average outcome for PR delivered by therapist 1 and the average outcome in the controls. The effect of pragmatic rehabilitation when delivered by therapist 2 is the difference in average outcome for PR delivered by therapist 2 and the average outcome in the controls. Hence the difference in efficacy between these two therapists at delivering PR is simply the difference between the corresponding average outcomes for the two therapists (the average outcome for the common controls drops out).

To identify whether there is a treatment by therapist interaction we examined whether there are differences in the therapist effects on outcome, both (i) separately for the two types of treatment, and (ii) for both types of treatment. These were evaluated simultaneously in a regression (ANCOVA) analysis including the interaction of therapist and treatment. The model was run for both primary outcomes, fatigue and physical functioning, at both outcome time points. In line with the trial’s original protocol and write-up, treatment effects were evaluated separately for the 20- and 70-week outcomes [5, 30].

We then used regression (ANCOVA) models to evaluate whether there were differences in the average patient-rated therapeutic alliance for the therapists. We compared the therapeutic alliance levels with the different therapists when they were delivering the two types of treatment, and as a treatment by therapist interaction. We evaluated whether the treatment effects for outcome were related to the therapist’s average patient-rated therapeutic alliance scores. The regression model was then repeated using only the task element of alliance to compare to the work of Heins *et al*. [27]. If a relationship between the treatment effects and the average patient-rated therapeutic alliance score for a therapist was found, a causal (instrumental variable) analysis [12] was planned.

In all of the regression models outlined in the preceding two paragraphs, in addition to the effect of type of intervention, the therapist and their statistical interaction, the regression models included, as additional covariates, baseline scores of whichever dependent variable was being regressed. Furthermore, the two variables which were used for stratification of the FINE trial sample, namely ambulatory status (whether or not the patient used a mobility aid on most days), and whether the London ME criteria [37]were met were also included in all of these regression models. These covariates were included to allow for chance imbalance, control for their effects and to increase precision; the effects of these covariates are not reported here. The model regressing patient-rated therapeutic alliance scores included the baseline fatigue score as a covariate to control for the effect of illness severity, but it did not include baseline therapeutic alliance as it is not meaningful to measure therapeutic alliance before the patient has met the therapist. Standard errors and 95% confidence intervals for parameters were calculated using robust (sandwich) estimators, allowing for the effects of possible skewness (lack of normality) in the data[38, 39].

A power calculation was conducted for each regression model using XLSTAT [40] in order the determine whether we had sufficient power to detect a moderate sized effect. The standardised effect size used was Cohen’s D which provides information about the size of the difference between two means, taking into account the variability in the data. A Cohen’s D of 0.25 is conventionally considered a moderate effect [41]. Our power calculation revealed that given the sample size and interactions, we have 0.91 power to detect a moderate effect size (Cohen’s D = 0.25) in the main intervention effects, and 0.84 power to detect a moderate effect size in the intervention by therapist interactions, with a 0.05 two-sided significance level. This research is adequately powered to detect a moderate effect size.

Stata 13.1 [42] was used for all other statistical procedures. Therapists can be modelled as fixed or random factors. In this report, therapists were modelled as fixed factors as (i) there were only three therapists, which is too small a sample to generalize to a population of therapists; (ii) the therapists were specially recruited and trained, and so not performing usual duties, and (iii) a mixed effects model would add unnecessary technical complexity. However, mixed models which treat therapists as random factors were also run to examine the sensitivity of the results to this type of model [43].

The trial had missing data in both the treatment outcomes and in measures of the therapeutic alliance. Our analyses considered the likely causes of missing data and how to address potential bias and lack of precision due to these missing data. The CALPAS had twenty-three percent missing data in the treated group. Patterns in the missing data were explored and multiple imputation (MI) was used to deal with potential bias and lack of precision due to missing data as CALPAS is an intermediate (not baseline or outcome) variable. The number of imputations should be at least the percentage of cases that are incomplete, so thirty imputations were added. For missing data in baseline variables, regression imputation was used before MI, as missing baseline values are independent of randomisation [44]. For missing outcome, weighting was used as MI is not appropriate for missing outcome data in trials [45, 46].

## Results

As previously reported [5] the 95 patients randomized to pragmatic rehabilitation had a mean age of 43.47 years (range 18–68), and 78% were female. The 101 patients randomised to supportive listening had a mean age of 45.13 years (range 21–68) and 79% were female. A fuller description of the patient samples is provided in the report of the main study [5]. Table 1 shows change in primary outcomes (fatigue and physical functioning) at weeks 20 and 70, as in the main trial findings [5] but here broken down by therapist. At the end of the treatment period, patients receiving PR had better outcomes than those receiving SL. Table 1 also shows patient-rated early alliance with each therapist for each therapy, measured at week one. The lowest patient-rated alliance was for therapist 3 when delivering SL.

**Table 1:** Mean (SD) changes in fatigue and physical functioning and therapeutic alliance, by therapy and by therapist.

	Pragmatic Rehabilitation mean (SD), % missing			Supportive Listening mean (SD), % missing		
	Therapist 1n = 34	Therapist 2n = 29	Therapist 3n = 32	Therapist 1n = 32	Therapist 2n = 35	Therapist 3n = 34
CALPAS Total	82.78 (14.42), 32.4	75.91 (23.80), 24.1	85.92 (17.68), 21.9	82.63 (14.33), 40.6	75.25 (14.64), 42.9	67.28 (18.63), 26.5
Post-MI CALPAS Total	82.28 (15.79), -	76.27 (23.03), -	85.52 (17.53), -	80.81 (14.99), -	75.97 (16.75), -	64.92 (19.49), -
CALPAS Task Element	63.81 (26.36), 34.4	53.33 (25.31), 31.5	46.90 (22.06), 14.7	69.13 (23.14)), 32.4	67.73 (30.54), 24.1	76.00 (22.55), 21.9
Post-MI CALPAS Task Element	64.98 (26.20), -	55.94 (25.89), -	45.40 (22.12), -	67.11 (23.75), -	67.12 (29.42), -	75.34 (22.50), -
*20 week outcomes*						
Δ Chalder Fatigue (scored 0011)	-2.63 (3.78), 8.8	-2.25 (3.53), 17.2	-1.26 (2.75), 15.6	-0.52 (2.40), 9.4	-0.81 (2.65), 5.7	-1.18 (3.06), 0.0
Δ Chalder Fatigue (scored 0123)	-7.53 (9.40), 8.8	-6.58 (7.65), 17.2	-5.78 (7.18), 15.6	-1.48 (6.52), 9.4	-2.84 (7.31), 5.7	-3.06 (6.29), 0.0
Δ SF-36 Physical Functioning Scale	+2.00 (3.93), 8.8	+2.52 (3.86), 13.8	+1.69 (4.29), 9.4	+0.34 (3.03), 9.4	+0.22 (4.66), 8.6	+0.85 (3.10), 2.9
*70 week outcomes*						
Δ Chalder Fatigue (scored 0011)	-1.67 (3.56), 14.7	-3.18 (4.27), 24.1	-0.80 (2.75), 21.9	-1.43 (3.44), 12.5	-1.14 (2.83), 17.1	-1.27 (3.16), 8.8
Δ Fatigue (scored 0123)	-6.15 (8.88), 14.7	-7.55 (8.20), 24.1	-3.20 (6.71), 21.9	-3.04 (8.63), 12.5	-3.50 (8.23), 17.1	-2.63 (7.23), 8.8
Δ SF-36 Physical Functioning Scale	+2.57 (4.01), 14.7	+3.50 (3.77), 17.2	+2.04 (3.77), 15.6	+1.17 (3.91), 9.4	+1.24 (5.04), 17.1	+0.69 (3.88), 5.9

^Δ^ represents change in score from baseline to outcome, calculated by subtracting baseline scores from outcome scores.

Reductions in the Fatigue scale indicate improvement. Increases in the SF-36 Physical Functioning scale indicate improvement. A higher CALPAS score indicates stronger alliance. All CALPAS measures are at week 1. ‘Post-MI’ means post multiple imputation.

### 1. Analysis of differences between therapists in the effects on outcome

Table 2 shows the results of the analysis of differences in therapist effects on outcome. The data were coded so that therapist 1 and SL are the comparison groups in the regression model. The first row of Table 2 (labelled therapist 2) is the therapist effect on fatigue (0011 scoring method) when therapist 2 delivered SL compared to when therapist 1 delivered SL. The second row is similarly defined. There are no significant therapist effects in the delivery of SL. The third row, labelled ‘therapy’, is the effect on outcome when therapist 1 delivered PR compared to when therapist 1 delivered SL. For therapist 1, PR was significantly more effective than SL at 20 weeks but not 70 weeks.

**Table 2:** Regression analyses of therapist effects on primary outcome measures.

	20 weeks				70 weeks			
Chalder Fatigue (scored 0011)	Coefficient	SE	P	95% CI	Coefficient	SE	p	95% CI
Therapist 2	-0.50	0.71	0.48	-1.90 to 0.91	0.38	0.89	0.67	-1.38 to 2.14
Therapist 3	-0.72	0.70	0.31	-2.10 to 0.66	0.25	0.90	0.78	-1.53 to 2.02
Therapy (effect of PR compared to SL in therapist 1)	-2.28	0.83	0.006	-3.91 to -0.65	-0.23	0.90	0.80	-2.00 to 1.54
Interaction of therapist 2 and PR	1.00	1.21	0.41	-1.39 to 3.40	-2.18	1.41	0.12	-4.96 to 0.61
Interaction of therapist 3 and PR	2.16	1.11	0.06	-0.03 to 4.35	0.76	1.20	0.53	-1.61 to 3.13
**halder Fatigue (scored 0123)**								
Therapist 2	-0.96	1.75	0.58	-4.42 to 2.49	0.13	2.21	0.95	-4.23 to 4.48
Therapist 3	-1.17	1.56	0.46	-4.24 to 1.91	0.89	2.09	0.67	-3.25 to 5.03
Therapy (effect of PR compared to SL in therapist 1)	-5.97	1.99	0.003	-9.90 to -2.03	-3.34	2.07	0.11	-7.44 to 0.75
Interaction of therapist 2 and PR	2.32	2.89	0.43	-3.40 to 8.03	-1.59	3.31	0.63	-8.12 to 4.93
Interaction of therapist 3 and PR	3.85	2.62	0.15	-1.34 to 9.03	3.37	2.82	0.23	-2.20 to 8.94
**SF-36 Physical Functioning Scale**								
Therapist 2	-0.21	0.91	0.82	-2.01 to 1.60	-0.10	1.12	0.93	-2.32 to 2.12
Therapist 3	0.49	0.73	0.50	-0.95 to 1.93	-0.56	0.90	0.54	-2.33 to 1.22
Therapy (effect of PR compared to SL in therapist 1)	1.80	0.87	0.04	0.08 to 3.51	1.10	1.15	0.34	-1.17 to 3.38
Interaction of therapist 2 and PR	0.19	1.42	0.90	-2.63 to 3.00	1.10	1.76	0.54	-2.38 to 4.58
Interaction of therapist 3 and PR	-1.47	1.26	0.24	-3.96 to 1.01	0.009	1.51	0.995	-2.98 to 3.00

Note that reductions in the Chalder Fatigue scale indicate improvement. Increases in the SF-36 Physical Functioning scale indicate improvement.

This analysis was repeated for fatigue (0123 scoring method) and physical functioning, as shown in Table 2. The results are consistent. Patients in receipt of PR fare significantly better than patients in receipt of SL at 20 weeks, but the difference is no longer significant at the 70 week outcome. In the main trial results [4], randomisation to PR only had a significant effect on the Chalder fatigue scale at 20 weeks when the fatigue scale was scored 0011. Readers should note that the analysis reported in this paper compares two therapies, delivered by different therapists, in contrast to the main trial results [4], which compared each therapy to GP treatment as usual. Therapist effects are not significant.

Simple one-way analysis of variance models provided estimates of the proportion of variance in outcomes which is associated with the therapist (intra-class correlations, see Table 3). For fatigue scored 0123 at 70 weeks, intraclass correlations were 0.10 and -0.10 for PR and SL, respectively. For physical functioning, there were intraclass correlations of 0.05 and -0.01 for PR and SL, respectively suggesting that differences between therapists accounted for little of the variance in outcomes.

**Table 3:** Intraclass Correlation Coefficients (ICCs) between ratings of therapeutic alliance (across three therapists) in the pragmatic rehabilitation and supportive listening conditions, and change in fatigue and physical functioning at 20 and 70 weeks.

	Pragmatic Rehabilitation Coefficient	Supportive Listening Coefficient
*20 week outcomes*		
Chalder Fatigue (scored 0011)	0.03	-0.08
Chalder Fatigue (scored 0123)	0.01	-0.07
SF-36 Physical Functioning Scale	0.02	-0.12
*70 week outcomes*		
Chalder Fatigue (scored 0011)	0.11	-0.38
Chalder Fatigue (scored 0123)	0.10	-0.10
SF-36 Physical Functioning Scale	0.05	-0.01

### 2. Analysis of differences in therapeutic alliance between therapists

Table 3 shows that there was a significant main effect of therapist and a significant interaction between therapist and therapy on the patient-rated CALPAS total score and on the CALPAS task element. The significant main effect is due to the lower level of patient-rated early therapeutic alliance when SL was delivered by therapist 3 compared to when SL was delivered by therapist 1. The significant interaction is the difference in level of therapeutic alliance when therapist 3 delivered PR compared to when therapist 1 delivered SL. Interpreting main effects in the presence of a statistically significant interaction requires caution. Inspecting the table of means (Table 1) reveals that for therapist 3, higher CALPAS values were obtained when delivering PR than when delivering SL. This accounts for both the main effect and interaction effect observed on the CALPAS scores. The results for the ‘task agreement’ element of therapeutic alliance exactly echo the results for the full therapeutic alliance, and have the same interpretation.

### 3. Is there any evidence of a relationship between TA and treatment effect?

Comparison of the significant effects in the regression models for the effects on outcome (Table 2) to the regression models for the therapeutic alliance (see Table 4) reveals that there is a different pattern of significant effects in the two models. This suggests that the component(s) which caused the significant difference in treatment effects (Therapist 1, PR vs SL) are not related to therapeutic alliance.

**Table 4:** Regression analyses of the effect of therapy and therapist on mean patient-rated therapeutic alliance. Models for both the CALPAS and CALPAS Task element are shown.

	CALPAS				CALPAS Task Element			
	Coefficient	SE	P	95% CI	Coefficient	SE	p	95% CI
Therapist 2	-8.47	4.93	0.09	-18.22 to 1.28	-10.40	7.26	0.15	-24.76 to 3.96
Therapist 3	-15.94	4.99	0.002	-25.82 to -6.06	-17.52	6.72	0.01	-30.81 to -4.22
Therapy (effect of PR compared to SL in therapist 1)	-0.72	4.63	0.88	-9.89 to 8.45	2.23	7.22	0.76	-12.08 to 16.54
Interaction of therapist 2 and PR	1.94	7.62	0.80	-13.14 to 17.02	10.38	10.39	0.32	-10.17 to 30.93
Interaction of therapist 3 and PR	18.91	7.03	0.008	5.00 to 32.82	24.24	9.41	0.01	5.62 to 42.85

Note that a higher CALPAS score indicates stronger alliance.

All analyses were repeated with mixed effects regression models which treat therapists as random factors. In addition, we ran regression models which were ‘complete case’ analyses; ignoring the missing data. Both of these supplementary sets of analyses gave results which were consistent with the models presented in this paper, and increased our confidence in the models presented.

## Discussion

We found no evidence that either therapist effects or therapeutic alliance significantly predicted changes in the two primary outcomes, fatigue and physical functioning, in a randomized controlled trial. The power calculation revealed that we had adequate power to detect effects of moderate size. Inspection of a table of correlations between therapeutic alliance and changes in outcomes, for each therapy and for each therapist at each assessment point revealed no consistent pattern of correlations (data in S1 Table). On this basis, it seems likely that the relationship between therapist effects, therapeutic alliance and outcome is not present in this study, rather than a lack of statistical power accounting for the results.

It is unusual, although not unknown, to find no therapist effects or effects of therapeutic alliance in trials of psychological or behavioural therapies. Perry and Howard (1989, cited in [17]) suggest therapist experience may be a critical factor, and that a sample of highly experienced therapists may yield significant variability on symptomatic outcome, whereas a sample containing less experienced therapists may yield more obvious therapist effects (up to 50% of outcome variance). The nurses used in the FINE trial were experienced primary care practitioners but prior to training were inexperienced in delivering therapy for CFS, and inexperienced at working within the confines of a research protocol. They were recruited specially to deliver the interventions in the FINE Trial, and all received identical training in both the delivery of the interventions and working to a protocol. Training involved supervised practice, role play and discussions, and the therapists received regular supervision during the time they were delivering the intervention. At the end of training all the therapists were considered competent to deliver the interventions. Fidelity to the treatment approaches was judged good by independent raters. It is possible therefore, that although prior to training we had three inexperienced therapists, at the end of training and during intervention delivery, we actually had three therapist who were closely matched for competence, and with similar approaches to patients. Our findings are in contrast to the findings of Wiborg *et al*.[20]. In Wiborg’s study, therapists were recruited from existing services. Therapist variation on symptomatic outcome was attributed to variation in therapists’ attitudes towards a manualised approach to treatment delivery. We did not measure therapists’ attitudes to the two interventions in the present study. Our therapists were not in the position of having to adjust prior practice to meet the demands of a research trial setting.

Godfrey *et al*. [22] observed significantly higher alliance scores when therapists delivered CBT compared to when delivering counselling. The authors, who used a fuller version of the CALPAS scale than the one we used, suggested that this may be due to an artefact of the CALPAS items selected, which included more technically than interpersonally focussed elements of therapy. The PACE trial [2] found no difference in the level of therapeutic alliance across treatment groups. In the FINE trial, the patient-rated early therapeutic alliance scores were, on average, higher when participants received PR (which has similarities to CBT), than when they received SL. However, this effect was only significant for therapist three. For therapists one and two, the differences in the mean alliance formed when delivering PR compared to SL were small. Comparison to other studies [2, 23, 27] suggested that the therapeutic alliance scores were typical for this patient group; meaning the results of the present study cannot be explained by unusually high or low alliances being formed.

No prior research in this area has randomized the allocation of therapist. The effects observed in prior research where patients were not randomized to therapists may be a mixture of treatment and selection effects. It is possible that in our study, by randomizing the therapist, we eliminated selection effects. Additionally, ours was a well-conducted study with adequate safeguards against sources of bias. Crits-Christoph and Mintz [19] found that studies with high quality control showed reduced therapist variation. It is possible that a combination of the use of high quality control and randomisation of therapists eliminated therapist effects in the present study. Whether it would be feasible or desirable to try to eliminate therapist effects in standard clinical practice is less clear. Attempts to standardise treatment delivery to more closely resemble the conditions of a controlled clinical trial might be difficult in clinical practice when therapists have a variety of priorities and calls on their time. Experienced therapists may not wish to alter aspects of their practice and mode of delivery of therapy which had worked for them over the years. It would be necessary to ensure that the most effective therapeutic techniques were combined with a standardised best therapist practice. Probably a more feasible approach would be to develop clearly defined and measurable therapist competencies[47] and to ensure through appointment procedures and clinical supervision that therapists were able to operate in accordance with these competencies. A further consideration is that, in a service with many patients and many therapists, there may be some scope for matching patients to therapists on individual characteristics, such as attachment style.

It may be the case that therapist effects and therapeutic alliance are of relatively limited importance in determining outcomes after treatment for chronic fatigue syndrome. There is growing evidence that the factors which mediate change after CBT and GET are changes in cognitive and behavioural factors such as symptom focusing, catastrophizing, and fear driven avoidance of activity[7, 9–11]. In the FINE Trial decreases in catastrophizing and self-reported activity limitation mediated change in fatigue following PR [11]. It is possible that these factors will change whenever treatments such as CBT, GET and PR are delivered competently. Supportive listening was not an effective treatment in the FINE Trial.

A key feature of this research was the random allocation of patients to therapists, thus excluding confounding factors such as location, or tendency to work with a particular subgroup of clients, in the analysis of therapist effects. This is a highly unusual feature of these data and a major strength. The sample size is large. Modern and statistically valid methods of dealing with missing data were used, including regression imputation for missing baseline data, multiple imputation for missing post-randomisation variables, and weighting for missing outcome data. Our confidence in our findings is increased by the fact that different statistical models all indicate the same results [43]. The standard deviation for the number of sessions attended is small (most individuals had close to the full treatment). In the SL arm, the mean number of sessions was 9.5 (S.D. 0.8), and in the PR arm, the mean number of sessions was 9.6 (S.D. 0.9) [5]. This reduces the effect of noncompliance from the randomized protocol in this analysis.

A limitation of the study is that the conclusion is potentially only valid for the range of recorded alliance levels, and that alliance was measured at the end of the first therapy session, which may have been too early for the therapeutic relationship to have formed. A small sample of only three therapists were used, which limits our ability to detect therapist effects, but is less important for the effect of therapeutic alliance on outcomes. While PR was effective in reducing fatigue in the FINE Trial, when compared with GP treatment as usual, neither PR nor SL significantly improved physical functioning, and there was little change in this outcome variable. This may reduce the likelihood of finding therapist effects or an effect of therapeutic alliance.

## Conclusions

The different therapists in the trial did not tend to form significantly different levels of alliance with clients, apart from therapist three when delivering PR compared to when delivering SL. Analysis of the outcomes did not indicate any significant therapist effects for change in physical functioning or fatigue at either 20 or 70 weeks; all therapists delivered both treatments at comparable levels of effectiveness. Furthermore, our analysis suggests that when specially trained nurse therapists delivered pragmatic rehabilitation or supportive listening at home to people with CFS/ME, the level of therapeutic alliance did not influence the effectiveness of treatment.

## Supporting Information

S1 Table Univariate correlations between therapeutic alliance and changes in fatigue and physical functioning by therapy and therapist. (DOCX)

## Abbreviations

CALPAS: California Psychotherapeutic Alliance Scale
CBT: cognitive behaviour therapy;
CFS: Chronic Fatigue Syndrome;
GET: graded exercise therapy;
ME: myalgic encephalomyelitis or encephalopathy;
PR: pragmatic rehabilitation;
SL: supportive listening;
TA: therapeutic alliance
