# Correction: Therapist Effects and the Impact of Early Therapeutic Alliance on Symptomatic Outcome in Chronic Fatigue Syndrome

**DOI:** 10.1371/journal.pone.0156120

**Published:** 2016-05-18

**Authors:** Lucy P. Goldsmith, Graham Dunn, Richard P. Bentall, Shôn W. Lewis, Alison J. Wearden

## Notice of Republication

S1 Dataset was published in error. The error was corrected in the XML and PDF versions of this article on May 9, 2016. Please download this article again to view the correct version.
