# Correction: Therapist Effects and the Impact of Early Therapeutic Alliance on Symptomatic Outcome in Chronic Fatigue Syndrome

**DOI:** 10.1371/journal.pone.0157199

**Published:** 2016-06-01

**Authors:** Lucy P. Goldsmith, Graham Dunn, Richard P. Bentall, Shôn W. Lewis, Alison J. Wearden

The dataset originally included as S1 Dataset was removed in consideration of possible restrictions for the public availability of the data related to the wording of the original consent form for the trial. Upon consultation with the authors’ university it has been established that the file may be publicly shared as it reports de-identified data. Please view S1 Dataset here.

The Data Availability statement for the article is revised to read: The authors have prepared a dataset that fulfills requirements in terms of anonymity and confidentiality of trial participants, and which contains only those variables which are relevant to the present study. Data are available as Supporting Information.

## Supporting Information

S1 Dataset De-identified trial data. (DTA)

## References

[1] Goldsmith LP, Dunn G, Bentall RP, Lewis SW, Wearden AJ (2015) Therapist Effects and the Impact of Early Therapeutic Alliance on Symptomatic Outcome in Chronic Fatigue Syndrome. PLoS ONE 10(12): e0144623 doi:10.1371/journal.pone.0144623 26657793 10.1371/journal.pone.0144623 PMC4685991

[2] Goldsmith LP, Dunn G, Bentall RP, Lewis SW, Wearden AJ (2016) Correction: Therapist Effects and the Impact of Early Therapeutic Alliance on Symptomatic Outcome in Chronic Fatigue Syndrome. PLoS ONE 11(5): e0156120 doi:10.1371/journal.pone.0156120 27249413 10.1371/journal.pone.0157199 PMC4889075

