# Supplementary material for: Therapist Effects and the Impact of Early Therapeutic Alliance on Symptomatic Outcome in Chronic Fatigue Syndrome
Source: PLoS One. 2015 Dec 14;10(12):e0144623. doi: 10.1371/journal.pone.0144623 (PMC4685991; doi:10.1371/journal.pone.0144623)
Supplement: S1 Table — (DOCX) [file pone.0144623.s001.docx]

**SI Table: Univariate correlations between therapeutic alliance and changes in fatigue and physical functioning, by therapy and therapist.**

|  | **Pragmatic** **Rehabilitation**  Pearson correlation, p | | | **Supportive Listening**  Pearson correlation, p | | |
| --- | --- | --- | --- | --- | --- | --- |
|  | Therapist 1 | Therapist 2 | Therapist 3 | Therapist 1 | Therapist 2 | Therapist 3 |
| *20 week outcomes* | | | | | | |
| ∆ Fatigue (scored 01) | -0.14,  p=0.52 | 0.31,  p=0.19 | 0.19,  p=0.37 | 0.34,  p=0.13 | -0.40,  p<0.05 | -0.42,  p=0.02 |
| ∆ Fatigue (scored 0123) | -0.07,  p=0.76 | 0.21, p=0.38 | 0.23,  p=0.28 | 0.18  p=0.44 | -0.12,  p=0.57 | -0.23,  p=0.23 |
| ∆ SF-36 | 0.31,  p=0.15 | 0.001,  p=0.995 | -0.41,  p=0.04 | -0.24,  p=0.28 | -0.03,  p=0.87 | -0.09,  p=0.62 |
| *70 week outcomes* | | | | | | |
| ∆ Fatigue (scored 01) | 0.32,  p=0.16 | 0.14,  p=0.56 | 0.32,  p=0.13 | -0.05,  p=0.83 | 0.44,  p=0.04 | 0.03,  p=0.88 |
| ∆ Fatigue (scored 0123) | 0.29  p=0.21 | 0.22  p=0.38 | 0.39  p=0.07 | 0.01  p=0.96 | 0.56,  p=0.007 | 0.24,  p=0.22 |
| ∆ SF-36 | 0.26,  p=0.25 | -0.27,  p=0.24 | -0.44,  p=0.04 | 0.06,  p=0.80 | 0.06,  p=0.80 | 0.06,  p=0.77 |

∆ represents ‘change in’ derived by subtracting baseline scores from outcome scores.
